# Unusual Triad of Unilateral Renal Agenesis, Ipsilateral Ureterocele, and a Blind-Ending Distal Ureter in an Adult: A Case Report

**DOI:** 10.7759/cureus.92847

**Published:** 2025-09-21

**Authors:** Venkata Ramana K, Nakul Aher, Lakshmi Kandhan L, Kalpana Ramachandran, Sriram Krishnamoorthy

**Affiliations:** 1 Urology, Sri Ramachandra Institute of Higher Education and Research, Chennai, IND; 2 Urology, Jupiter Hospital, Thane, IND; 3 Anatomy, Sri Ramachandra Institute of Higher Education and Research, Chennai, IND

**Keywords:** blind-ending ureter, genitourinary anomalies, lower urinary tract symptoms, renal agenesis, ureterocele

## Abstract

In adults, a ureterocele is a solitary, intravesical abnormality with the normal ureteral insertion at the vesicoureteral junction. We present the case of a 47-year-old man with symptoms of mixed voiding and storage lower urinary tract symptoms, demonstrating the unusual combination of ureterocele, renal agenesis, and a blind-ending ureter on the ipsilateral side, as well as its clinical manifestation and treatment. The right-sided renal agenesis and ureterocele were incidentally found on imaging during the workup. A magnetic resonance imaging (MRI) scan confirmed that the right kidney was absent, which was not seen by an ultrasound, and also noted a ureterocele. Cystoscopy confirmed the diagnosis of the ureterocele. The blind-ending distal ureter was noted during retrograde pyelography, and an endoscopic incision was successfully performed, after which the patient recovered well. Our case report gives more importance to the existence of these anomalies and the understanding of their embryological basis. The knowledge clinicians possess regarding ureterocele in adults with renal agenesis will help them be suspicious of patients presenting with lower urinary tract symptoms or frequent urinary tract infections. Early imaging and endoscopic treatment are crucial to prevent complications, including contralateral renal damage, and improve outcomes.

## Introduction

Ureterocele in adults is usually reported as an isolated urinary tract abnormality with the normal ureteral insertion at the vesicoureteral junction [1]. Endoscopic procedures are less invasive and are successful in symptom relief, as ureteroceles in adults are intravesical and less obstructive. About 80% of ureteroceles are linked to a duplex collecting system's upper pole; intravesical ureteroceles are more prevalent in single systems [2]. Ureterocele and its association with congenital anomalies are well known, but the concomitant presence of ureterocele, a blind-ending ureter, and renal agenesis is a rare occurrence. Ureteroceles in adults are usually isolated and intravesical. In children, they are often linked to duplex systems, particularly the upper pole, and may insert ectopically (urethra, vagina, vestibule, perineum), causing leakage or incontinence. Incompetence of a rudimentary ureteric orifice can lead to vesicoureteral reflux into a blind-ending or dysplastic ureter, explaining dilation despite absent renal function. They are classified as intravesical (orthotopic, less obstructive) or extravesical/ectopic (associated with duplex systems, obstruction, or incontinence) [3]. The literature revealed just six previously reported cases describing the concurrence of all three features. This study aims to contribute to the existing knowledge of genitourinary anomalies to improve patient safety by highlighting the clinical and embryological significance of ureterocele and renal agenesis in patients who may present with lower urinary tract symptoms in adult life.

The presence of ureteroceles in patients with unilateral renal agenesis has important embryological implications and clinical relevance, and various theories have been proposed to explain this association. The synchronous occurrence of these two anomalies suggests a possible defect in the development of the ureteric bud during embryogenesis, which may ultimately result in renal agenesis. Another proposed explanation is the theory of supernumerary ureteric buds, in which multiple buds may form, leaving behind remnants such as blind-ending ureters or ureteroceles even in the absence of a functioning kidney. Additionally, the failure of Chwalla's membrane perforation has been implicated; under normal circumstances, this transient membrane at the ureterovesical junction dissolves to permit urine flow, but in certain cases, partial persistence or incomplete perforation can obstruct the ureter, leading to ureterocele formation despite the lack of a functional kidney [4].

A ureterocele in the absence of a functional kidney raises important questions about its etiology, with one possible explanation being the accumulation of epithelial secretions within the blind-ending ureter, leading to progressive dilation and eventual formation of the ureterocele. Despite being non-secretory and non-functional, a ureterocele can still cause clinical problems such as bladder outlet obstruction and lower urinary tract symptoms, making it important to consider this anomaly in patients presenting with unexplained lower urinary tract symptoms. In addition, an obstructing and enlarged ureterocele may result in bladder dysfunction over time, which in turn can cause vesicoureteral reflux and subsequent damage to the contralateral kidney. This underscores the need for prompt diagnosis and early treatment of such ureteroceles to avoid collateral damage [5].

## Case presentation

**Figure 1:**
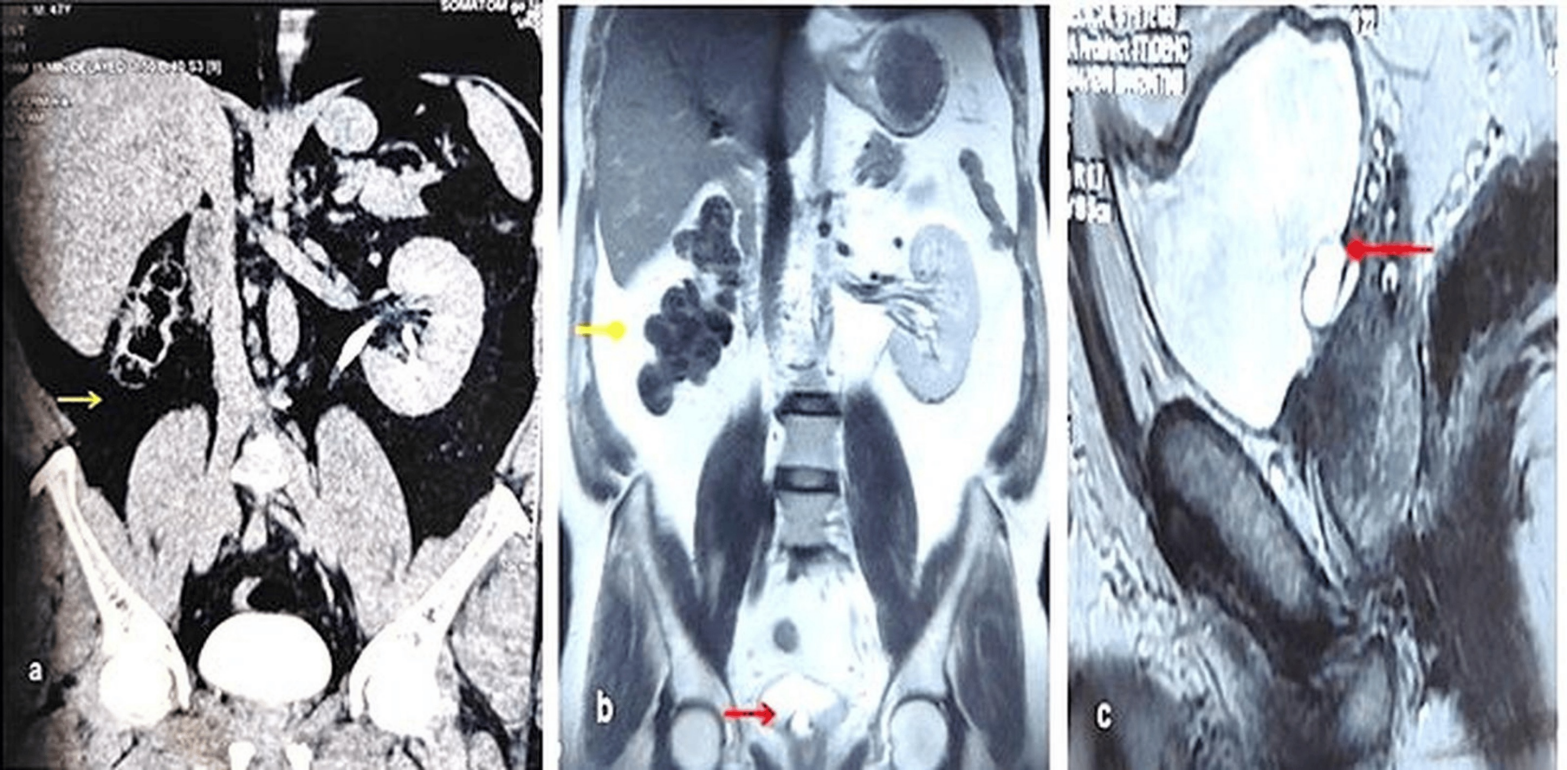
CT urography and MRI findings (a) CT urography findings showing an empty right renal fossa (yellow arrow). (b and c) Right renal agenesis (yellow arrow) with a right ureterocele (red arrow) CT: computed tomography; MRI: magnetic resonance imaging

A 47-year-old male patient arrived at the urology department in March 2025 with complaints of mixed voiding and storage lower urinary tract symptoms. Hematological and biochemical investigations and urine routine microscopy reports were normal. Clinical examination was normal. The patient was evaluated with an ultrasound of the abdomen, which revealed an absent right kidney in the right renal fossa and a right-sided ureterocele in the urinary bladder. Computed tomography (CT) urography was performed to further evaluate the entire urinary tract. The CT study showed an absent right kidney with a normally functioning left kidney (Figure 1a). The left ureter was normal. Interestingly, no definitive evidence of a ureterocele was identified within the urinary bladder on imaging.

**Figure 2:**
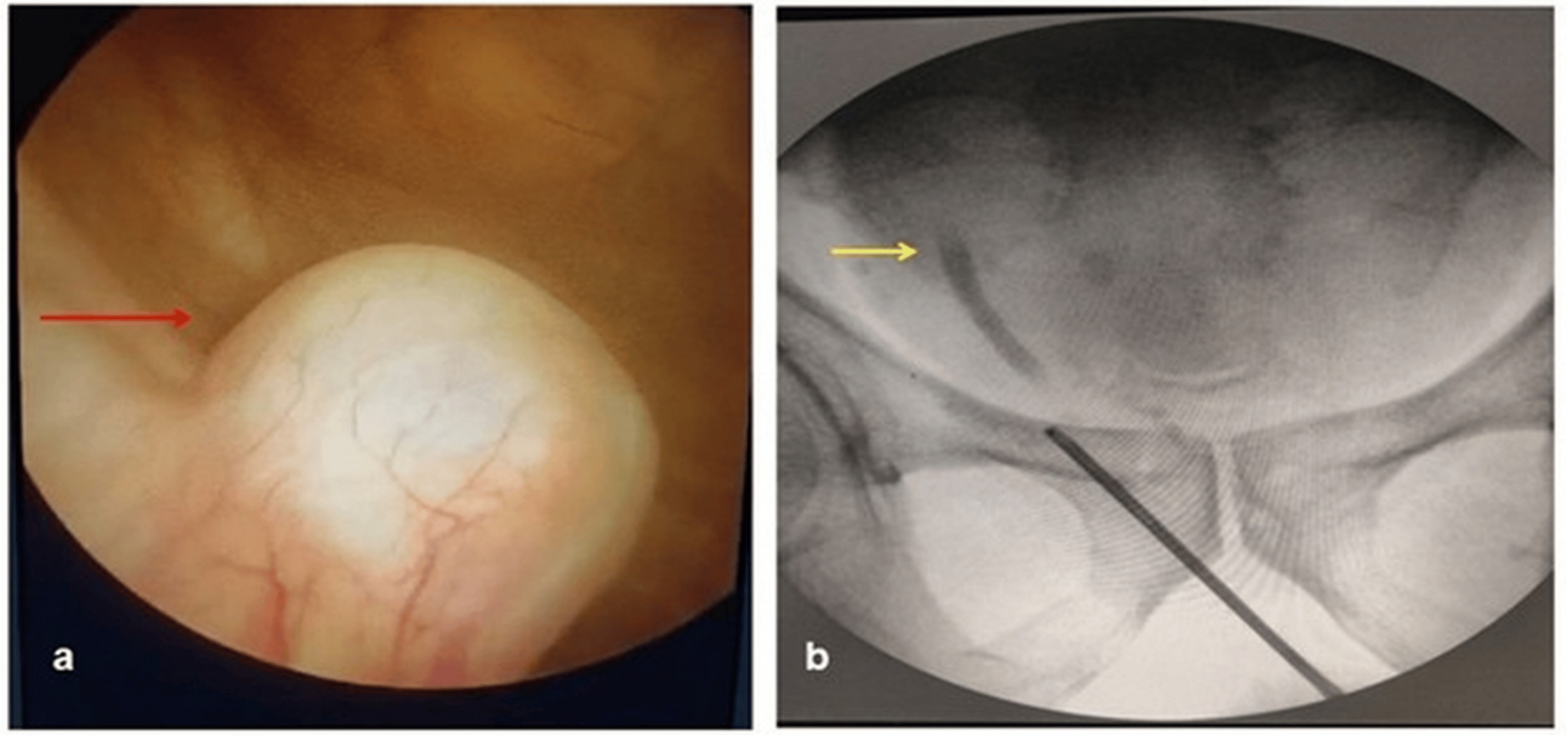
Cystoscopy and RGP findings (a) Cystoscopy showing a right ureterocele (red arrow). (b) Intraoperative RGP illustrating a blind-ending right distal ureter (yellow arrow) RGP: retrograde pyelography

Office cystoscopy was performed, and the presence of a right ureterocele was confirmed (Figure 2).

Magnetic resonance imaging (MRI) was performed to rule out the presence of an ectopic (pelvic) kidney. The MR images demonstrated right renal agenesis. The proximal and mid-right ureter was not visualized, with the distal-most part showing focal cystic dilation at the right vesicoureteral junction, representing a ureterocele (Figure 1b-1c). Intraoperatively, retrograde pyelography was done, showing a blind-ending distal ureter.

An endoscopic incision of the ureterocele was done. During the postoperative period, the patient recovered well uneventfully and was discharged on the first postoperative day. At the three-month follow-up, the patient remained asymptomatic, with the complete resolution of lower urinary tract symptoms, and continues to be on regular follow-up.

## Discussion

Anomalies comprising renal agenesis are not uncommon; however, it is uncommon for renal agenesis, ureterocele, and a distal ureter with a proximal blind end on the ipsilateral side to occur together.

The widely recognized explanation for the development of ureteroceles is that Chwalla's membrane partially dissolves during embryogenesis, obstructing the ureteral opening [6]. Normally, the membrane bursts, allowing urine to flow freely. Chwalla's membrane causes the distal ureter (the part inside the urogenital sinus) to grow since it is located inside the developing bladder's muscles. Ureterocele is the result of the ureter's distal expansion inside the bladder.

A non-functioning multicystic dysplastic, dysplastic, or hypoplastic kidney may have early involution as a secondary cause of a missing kidney or a true case of renal agenesis [7]. When the ureteric bud fails to develop and hence fails to cause the differentiation of the metanephrogenic mesenchyme to the renal tubular epithelium, true renal agenesis (as well as many other renal abnormalities) develops. Nephrons and the metanephric system's collecting system are formed due to organogenesis induced by the ureteric epithelium. The coexistence of unilateral renal agenesis, ipsilateral ureterocele, and a blind-ending ureter, particularly in an adult, represents a rare and noteworthy clinical association. These structures are believed to develop from either supernumerary ureteric buds or an abnormal connection between the ureteric bud and the metanephrogenic cap [8]. Hence, as this case draws attention to the association of ureterocele in the absence of an ipsilateral kidney, without any pooling of urine to attribute to the dilation of the distal ureter, it opens a channel for research about the embryological and etiological aspects of ureterocele. The development of a ureterocele when a kidney is not present may be accounted for by the secretions from the blind-ending ureter that accumulated over time. We would like to propose a hypothesis that the collection of the secretions of the epithelium due to changes in the epithelium of the blind-ending ureterocele over the years might be the cause of the ureterocele here. There is a possibility that vesicoureteral reflux into a rudimentary or blind-ending ureter may explain the formation of the ureterocele in our case. Although CT imaging did not clearly demonstrate reflux, intraoperative findings of a blind-ending distal ureter with ureterocele support this mechanism as a plausible theory. 

Our patient had renal agenesis and showed signs of ureterocele-related urinary tract blockage. The concomitant presence of ureterocele, renal agenesis, and a blind-ending distal ureter is extremely rare. Table 1 enlists previously reported cases with all three concurrent characteristic anomalies.

**Table 1:** Previously published reports on the concomitant presence of ureterocele, renal agenesis, and a blind-ending distal ureter in adults LUTS: lower urinary tract symptoms; UTI: urinary tract infection

Author	Age at presentation, gender	Laterality	Presentation	Management
Bhayana and Jain [9]	20 years old, male	Right	Voiding LUTS, lower abdominal pain	Not available
Mohseni et al. [10]	32 years old, male	Left	Fever with a pelvic mass	Laparoscopy followed by open ureterectomy with the resection of the retrovesical pyo-ureterocele
Ahmed [11]	30 years old, male	Left	Left lower abdominal pain, recurrent UTI	Open resection of the megaureter stump and ureterocele
Berg et al. [12]	38 years old, male	Right	-	Open resection of the ureter stump and ureterocele with simultaneous vesiculectomy
Aravind et al. [13]	61 years old, male	Left	Left gluteal region pain	Robot-assisted laparoscopic excision of the ureteric remnant
Our case	47 years old, male	Right	Mixed voiding and storage LUTS	Endoscopic incision of the ureterocele

**Figure 3:**
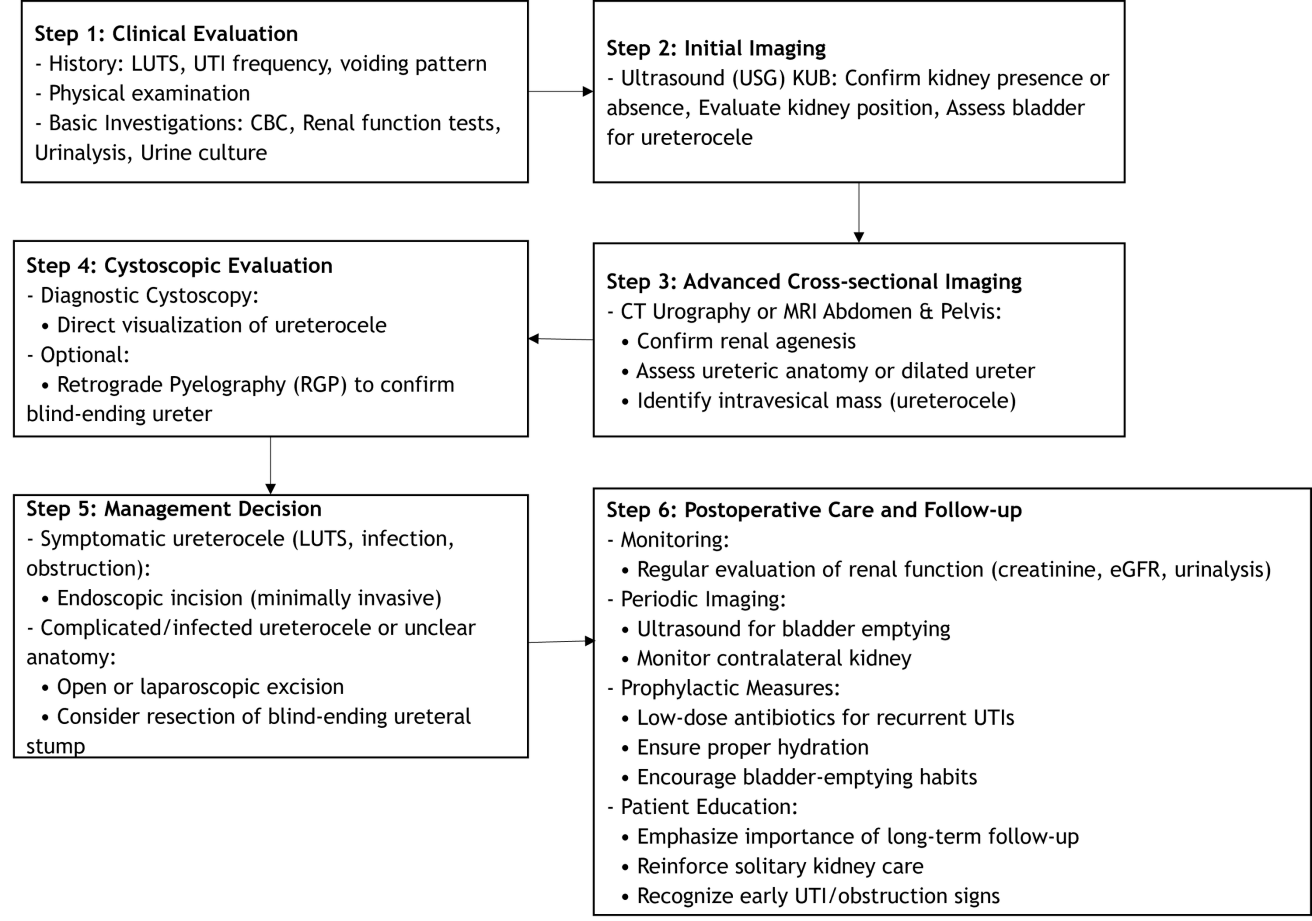
Clinical approach for the evaluation and management of ureterocele with renal agenesis LUTS: lower urinary tract symptoms; UTI: urinary tract infection; CBC: complete blood count; KUB: kidneys, ureters, and bladder; CT: computed tomography; MRI: magnetic resonance imaging; eGFR: estimated glomerular filtration rate Image credit: Author's own illustration

If a patient with renal agenesis presents symptoms related to the lower urinary tract or recurrent urinary tract infections, clinicians should suspect a ureterocele. In such circumstances, initiating the investigation as soon as possible is imperative. An endoscopic intervention could potentially spare the contralateral urinary system from both ascending retrograde infection and obstruction induced by the ureterocele. The clinical approach for the evaluation and management of ureterocele with renal agenesis is depicted in Figure 3.

Take-home messages

Each of the congenital anomalies can appear in isolation, but unilateral renal agenesis and ipsilateral ureterocele with a blind-ending distal ureter in one patient is extremely rare. Clinicians must look for such congenital anomalies while evaluating lower urinary tract symptoms.

Ureterocele in the absence of an ipsilateral renal unit warrants detailed evaluation to look for an embryological explanation. An abnormal course of ureteric bud development or the presence of supernumerary ureteric buds needs to be looked for. Secretions from the blind-ending ureter may contribute to ureterocele formation over time due to chronic accumulation.

Early and comprehensive imaging is needed to identify such complex urological anomalies. Renal agenesis and ureterocele formation were incidentally detected, which led to timely intervention. A high index of clinical suspicion is needed in such patients to facilitate early detection and prompt treatment.

## Conclusions

The presence of an ipsilateral ureterocele in unilateral renal agenesis suggests an underlying embryological defect and has significant clinical implications. It emphasizes the need for early suspicion, prompt imaging, and appropriate intervention to prevent bladder dysfunction and protect the remaining functional kidney.
